# Antineoplastic effects and mechanisms of micheliolide in acute myelogenous leukemia stem cells

**DOI:** 10.18632/oncotarget.11342

**Published:** 2016-08-17

**Authors:** Qing Ji, Ya-hui Ding, Yue Sun, Yu Zhang, Hui-er Gao, He-nan Song, Ming Yang, Xiao-lei Liu, Zi-xiang Zhang, Ying-hui Li, Ying-dai Gao

**Affiliations:** ^1^ State Key Laboratory of Experimental Hematology, Institute of Hematology and Hospital of Blood Diseases, Chinese Academy of Medical Sciences and Peking Union Medical College, Tianjin 300020, P. R. China; ^2^ The State Key Laboratory of Medicinal Chemical Biology, College of Pharmacy, Nankai University, Tianjin 300071, P. R. China; ^3^ Department of Stomatology, Institute of Hematology and Blood Diseases Hospital, Chinese Academy of Medical Sciences and Peking Union Medical College, Tianjin 300020, P. R. China

**Keywords:** leukemic stem cells, micheliolide

## Abstract

Leukemic stem cells (LSCs) greatly contribute to the initiation, relapse, and multidrug resistance of leukemia. Current therapies targeting the cell cycle and rapidly growing leukemic cells, including conventional chemotherapy, have little effect due to the self-renewal and differentiated malignant cells replenishment ability of LSCs despite their scarce supply in the bone marrow. Micheliolide (MCL) is a natural guaianolide sesquiterpene lactone (GSL) which was discovered in michelia compressa and michelia champaca plants, and has been shown to exert selective cytotoxic effects on CD34^+^CD38^−^ LSCs. In this study, we demonstrate that DMAMCL significantly prolongs the lifespan of a mouse model of human acute myelogenous leukemia (AML). Mechanistic investigations further revealed that MCL exerted its cytotoxic effects via inhibition of NF-κB expression and activity, and by generating intracellular reactive oxygen species (ROS). These results provide valuable insight into the mechanisms underlying MCL-induced cytotoxicity of LSCs, and support further preclinical investigations of MCL-related therapies for the treatment of AML.

## INTRODUCTION

The presence of leukemic stem cells (LSCs) has been confirmed in several human hematological malignancies, and is considered as a key factor that contributes to the initiation, maintenance, relapse, and drug-resistance of leukemia [1, 2]. Similar to normal hematopoietic stem cells (HSCs), LSCs have the capacity to self-renew and differentiate, thereby allowing them to propagate and generate additional progeny with defective development potential. Consequently, hematopoietic homeostasis is compromised and the risk of leukemia and other hematological malignancies are enhanced [3, 4]. Conventional chemotherapy eliminates the bulk of rapidly growing, differentiated leukemic cells, yet it fails to completely eradicate the significantly smaller pool of LSCs that is often present as a relatively quiescent cell population [5, 6]. Therefore, novel LSC-directed therapies that specifically target this rare stem cell fraction are urgently needed to combat leukemia.

LSCs exhibit similar stem-like characteristics to HSCs, although the unique features that render LSCs as a potential risk factor of hematological malignancies remain unknown. It is hypothesized that LSCs rely on cell-intrinsic signaling pathways. Several canonical developmental pathways, including the Wnt/β-catenin and Hedgehog signaling pathways which are evolutionarily conserved and tightly controlled in adult tissues, play an essential role in normal stem cell function [7, 8]. Previous studies have shown that the activation of β-catenin in LSCs enhances self-renewal activity and leads to abnormal expansion and leukemic potential [9, 10]. Additionally, the PI3K/AKT/mTOR pathway plays a major survival role during normal hematopoietic homeostasis, yet is frequently dysregulated in leukemia [11–13]. Currently, abnormal expression of NF-κB is regarded as a crucial contributor to leukemogenesis, and targeted therapies involving NF-κB have been designed to induce LSCs-specific apoptosis [14]. Two regulators of NF-κB that have been implicated as tumor suppressor genes, DAPK and IRF-1, have been found to be deregulated and present in many samples obtained from patients with acute myelogenous leukemia (AML) [15]. Higher levels of reactive oxygen species have also been observed in primitive leukemic cells compared to normal lymphocytes [16, 17]. Furthermore, the adaptation of leukemic cells to oxidative stress is considered to be a significant factor in establishing a drug resistance phenotype [18]. Therefore, elucidation of the mechanistic details of the key signaling pathways that modulate LSC proliferation and self-renewal will be increasingly critical not only for the generation of LSC-targeted therapies, but also for a better understanding of the pathological mechanisms underlying disease initiation and progression.

Small molecules offer great advantages for the research and treatment of leukemia due to their rapid and diverse effects on many different cell types. Several small molecules, such as parthenolide (PTL), impart specific cell-killing effects on LSCs. For example, inhibition of NF-κB and production of reactive oxygen species (ROS) appear to be essential for PTL-mediated activity against CD34^+^CD38^−^ LSCs [19, 20]. However, the instability of PTL under acidic and basic conditions limits its application as a molecular probe *in vivo* or as a therapeutic reagent [21]. We previously characterized micheliolide (MCL), a natural guaianolide sesquiterpene lactone (GSL) from Michelia compressa and Michelia champaca plants [22]. We also synthesized MCL from PTL [22]. MCL was found to selectively eradicate AML stem/progenitor cells (e.g., CD34^+^CD38^−^ cells). MCL and its water-soluble Michael adduct, DMAMCL, have exhibited strong anti-inflammatory properties [23]. Furthermore, DMAMCL displayed higher plasma stability, a more sustained release, and superior *in vivo* efficacy compared to DMAPTL [24]. The goal of this study was to characterize the potential for MCL to serve as an LSC-targeted therapy. Therefore, the effects of MCL on different hematopoietic cell populations from patients with AML, and also in a humanized mouse model of leukemia, were investigated. Furthermore, we sought to elucidate the mechanisms by which MCL functions to selectively induce apoptosis in LSCs.

## RESULTS

### MCL inhibits cell proliferation and selectively induces apoptosis in leukemic stem/progenitor cells (LSPCs)

**Figure 1:**
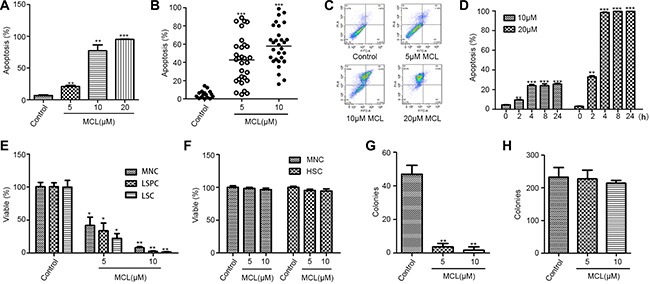
MCL selectively induces the apoptosis of AML leukemic stem cells, but not normal hematopoietic stem cells (**A**) Percentage of apoptosis was assessed in KG1a cells treated with MCL. Control represents untreated cells. (**B**) Percentage of apoptosis was assessed in cells isolated from primary AML specimens (*n* = 20) and treated with MCL. (**C**) Representative flow cytometry scatter plots displaying apoptotic cell populations after treatment with MCL. (**D**) Percentage of apoptosis was assessed in KG1a cells treated with MCL at different time points over 24 h. (**E**) Percentage of viability was assessed in MNCs, LSCs (CD34^+^), and LSPCs (CD34^+^ CD38^−^) isolated from AML specimens and treated with MCL. (**F**) Percentage of apoptosis was assessed in MNCs and HSCs (CD34^+^CD38^−^) isolated from human umbilical cord blood and treated with MCL. (**G**) Colony-forming abilities of MNCs isolated from primary AML specimens and treated with MCL. (**H**). Colony-forming abilities of HSCs isolated from human umbilical cord blood and treated with MCL. Control represents untreated cells. Error bars represents the SEM. ***P < 0.001; **P < 0.01; *P < 0.05; ns, no significance.

We previously demonstrated that MCL inhibits leukemia cell proliferation. Accumulating data suggest that cells that hyper-express of multi-drug resistance related genes show the phenotype CD34+ in human hematopoietic stem cells. It has been reported that multi-drug resistance related genes may be involved in the regulation of key processes of stem cells [25]. Therefore, we first assessed whether MCL could also inhibit the proliferation of drug-resistant leukemic cell lines. Meanwhile, KG1a cells exhibit an LSC-like phenotype with high levels of CD34 expression (98.6%) and low levels of CD38 expression (24.6%), so it was considered as a leukemia stem-like cell line. When drug-resistant cell lines were treated with MCL, significant cytotoxicity was induced in leukemia multi-drug resistant cells (Table 1). In particular, increasing concentrations of MCL induced apoptosis in KG1a cells (Figure 1A and 1C) and primary AML cell (Figure 1B) in a dose-dependent manner. In a time course assay, high levels of apoptosis were detected 2 h after MCL treatment. The level of apoptosis further increased after 4 h, and then remained constant up to 24 h later in KG1a cell line (Figure 1D). Thus, MCL induced significant cytotoxicity in multidrug-resistant leukemic cells for at least 24 h.

**Table 1:** Cytotoxicity of multidrug-resistant cell lines exposed to MCL or ADR

Cell Line	MCL (SD ± SEM, μM)	ADR (SD ± SEM, μM)
HL60	4.30 ± 0.02	0.0165 ± 0.0078
HL60/ADR	6.25 ± 1.06	4.85 ± 1.06
K562	7.85 ± 1.48	0.06 ± 0.01
K562/A02	8.15 ± 0.49	8.55 ± 2.19
K526/G	5.15 ± 0.49	0.028 ± 0.011
KG1a	9.40 ± 0.28	0.72 ± 0.34

All IC_50_ values are the average of three independent experiments; ADR: adriamycin was used as a positive control.

Twenty primary AML specimens were subsequently selected to investigate the effects of MCL on different hematopoietic cell populations. CD34^+^ LSPCs and total mononuclear cells (MNCs) were sorted and isolated from tissue samples. Treatment of the LSPCs with MCL induced greater cytotoxicity compared to the treatment of total MNCs (Table 2 and Figure 1E). In contrast, MCL treatment did not induce significant cytotoxic effects in the hematopoietic stem and progenitor cells (HSPCs) obtained from human umbilical cord blood (Figure 1F).

**Table 2:** Viability of primary AML cells treated with MCL

AML Sample	subtype	Total Cells (% viable)		LSPCs (% viable)	
		5 μM MCL	10 μM MCL	5 μM MCL	10 μM MCL
AML1	M3	88.0	80.0	56.5	53.4
AML2	M6	70.1	47.3	88.2	73.9
AML3	CML-AP	78.0	59.0	65.7	23.5
AML4	M4	17.4	15.4	11.0	9.0
AML5	M5	45.3	27.7	34.7	23.9
AML6	M2	93.7	78.7	95.3	74.2
AML7	N/A	73.3	58.2	65.9	53.7
AML8	N/A	79.8	61.9	65.7	31.2
AML9	M3	29.1	11.9	14.5	3.7
AML10	M1	48.9	35.1	38.7	11.5
AML11	M4	67.2	42.1	23.0	21.2
AML12	M2	42.2	36.7	23.8	13.4
AML13	M4	11.9	3.2	10.0	1.1
AML14	M3	56.0	54.6	50.3	35.2
AML15	M2	89.0	42.1	93.0	45.3
AML16	M3	77.1	58.0	56.5	31.3
AML17	M5	58.0	43.5	55.7	46.0
AML18	M4	34.1	8.9	29.0	2.8
AML19	M5	28.7	18.7	34.7	21.6
AML20	M5	49.9	35.0	23.6	16.3

All values were carried out as triplicated. And the percentage of viable was compared as its control.

To further characterize the effects of MCL on leukemic cells, methylcellulose colony-forming unit (CFU) assays were performed. The number of colonies was significantly reduced in response to MCL treatment compared to untreated controls (Figure 1G). Notably, cells isolated from AML1, AML4, and AML10 tissue specimens failed to form any colonies after treatment with MCL (Table 3). Conversely, MCL treatment of HSPCs obtained from human umbilical cord blood had no effect on colony formation, independent of MCL concentration (Figure 1H). Taken together, these data suggest that MCL specifically inhibits malignant hematopoiesis of LSPCs, and not normal hematopoiesis.

**Table 3:** Colony formation of primary AML cells treated with MCL

AML Sample	Number of colonies (SD ± SEM, μM)		
	Control	5 μM MCL	10 μM MCL
AML1	36.0 ± 8.5	0	0
AML2	50.0 ± 5.2	2.7 ± 1.5	0.3 ± 0.6
AML3	47 ± 5.3	3.7 ± 2.1	1.7 ± 2.1
AML4	10.0 ± 2.6	3.7 ± 1.5	0
AML6	222.0 ± 41.0	158.0 ± 24.0	2.3 ± 4.0
AML7	45.0 ± 13.7	37.0 ± 4.2	4.7 ± 4.2
AML8	114.3 ± 20.5	44.7 ± 16.9	7.0 ± 6.0
AML10	26.0 ± 2.8	0	0

All values were carried out as triplicated. And each test was tested for three times.

### MCL improves survival in a mouse model of human AML

The finding that MCL eliminated LSPCs *in vitro* led us to further analyze the effect of MCL in an *in vivo* system. A NOD/SCID xenotransplantation leukemia model was established following irradiation (250 cGy) and an injection of primary human AML MNCs (1 × 10^7^). CD45^+^ cells were detected in the bone marrow 8 weeks later, indicating that a successful engraftment had occurred to generate AML mice. The pro-drug form of MCL, DMAMCL, was orally administered to these AML mice at doses of 25 mg/kg, 50 mg/kg, and 100 mg/kg for a total of 7 treatments every other day. As a positive control, ADR, a traditional chemotherapy drug, was injected via the tail vein at a dose of 2 mg/kg for a total of 4 treatments every two days.

**Figure 2:**
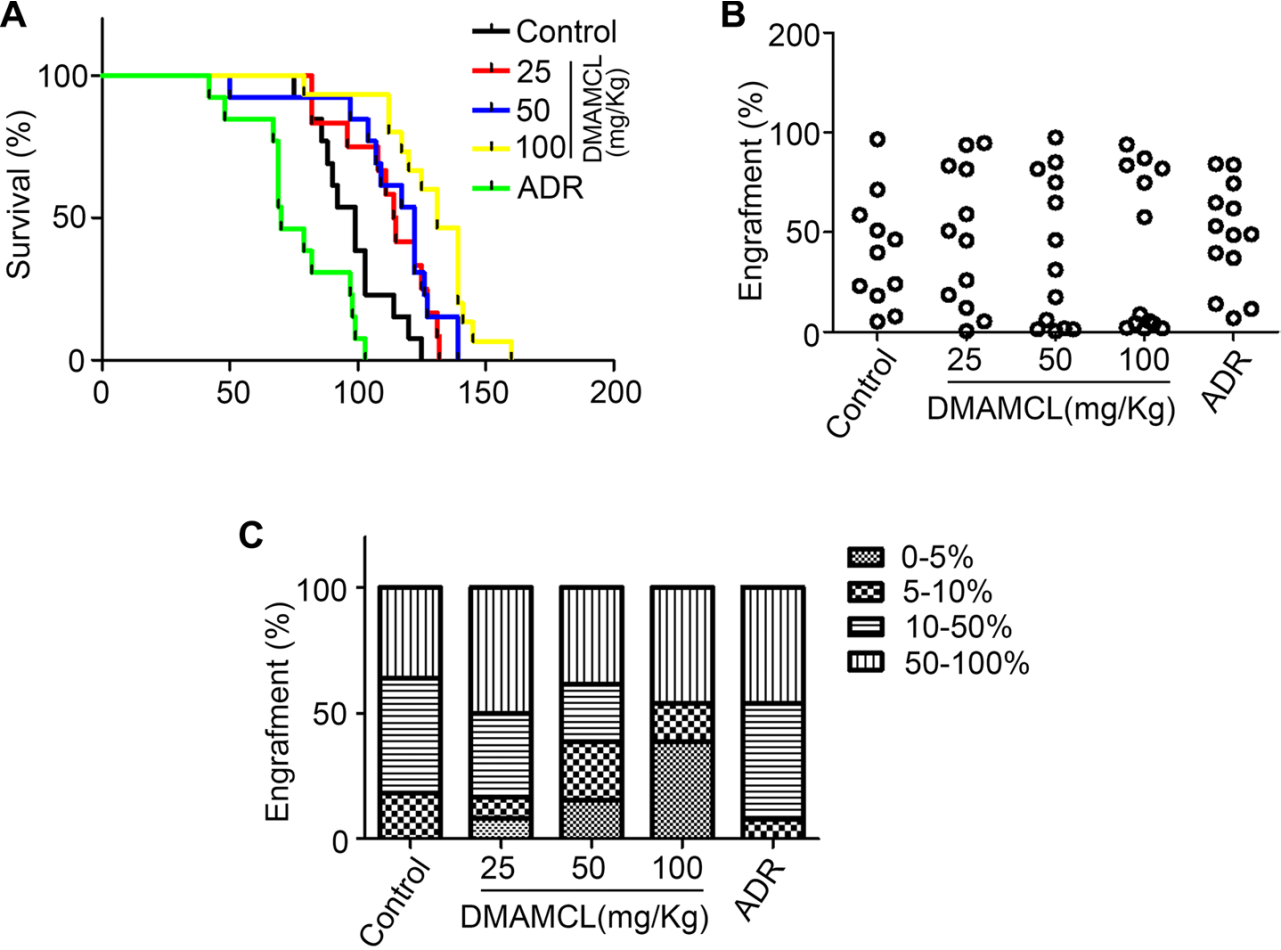
MCL improves the survival of mice with human AML (**A**) Survival plot representing the percentage of surviving NOD/SCID mice injected with human AML cells and treated with varying doses of DMAMCL. (**B**) Percentage of CD45^+^ cell engraftment in the bone marrow of mice after treatment with control, DMAMCL, or ADR. (**C**) Percentage of mice exhibiting varying degrees of leukemic cell engraftment after treatment with control, DMAMCL, or ADR. Administration of PBS is represented by Control and ADR was administered as a positive control.

The average lifespan of the AML model was markedly increased compared to the control group which was injected with PBS (Figure 2A). CD45^+^ cell engraftment was then determined by flow cytometry (Figure 2B). A greater number of mice with lower level of engraftment (< 5%) were observed after DMAMCL treatment (Figure 2C). Meanwhile, the number of mice exhibiting engraftment levels at 5–10% and 10–50% after DMAMCL treatment was reduced (Figure 2C). Collectively, these results suggest that MCL treatment improved the survival of the human AML mouse model.

### Transcriptome analysis of MCL-treated leukemic cells reveals differential expression of genes associated with ROS and NF-κB signaling

**Figure 3:**
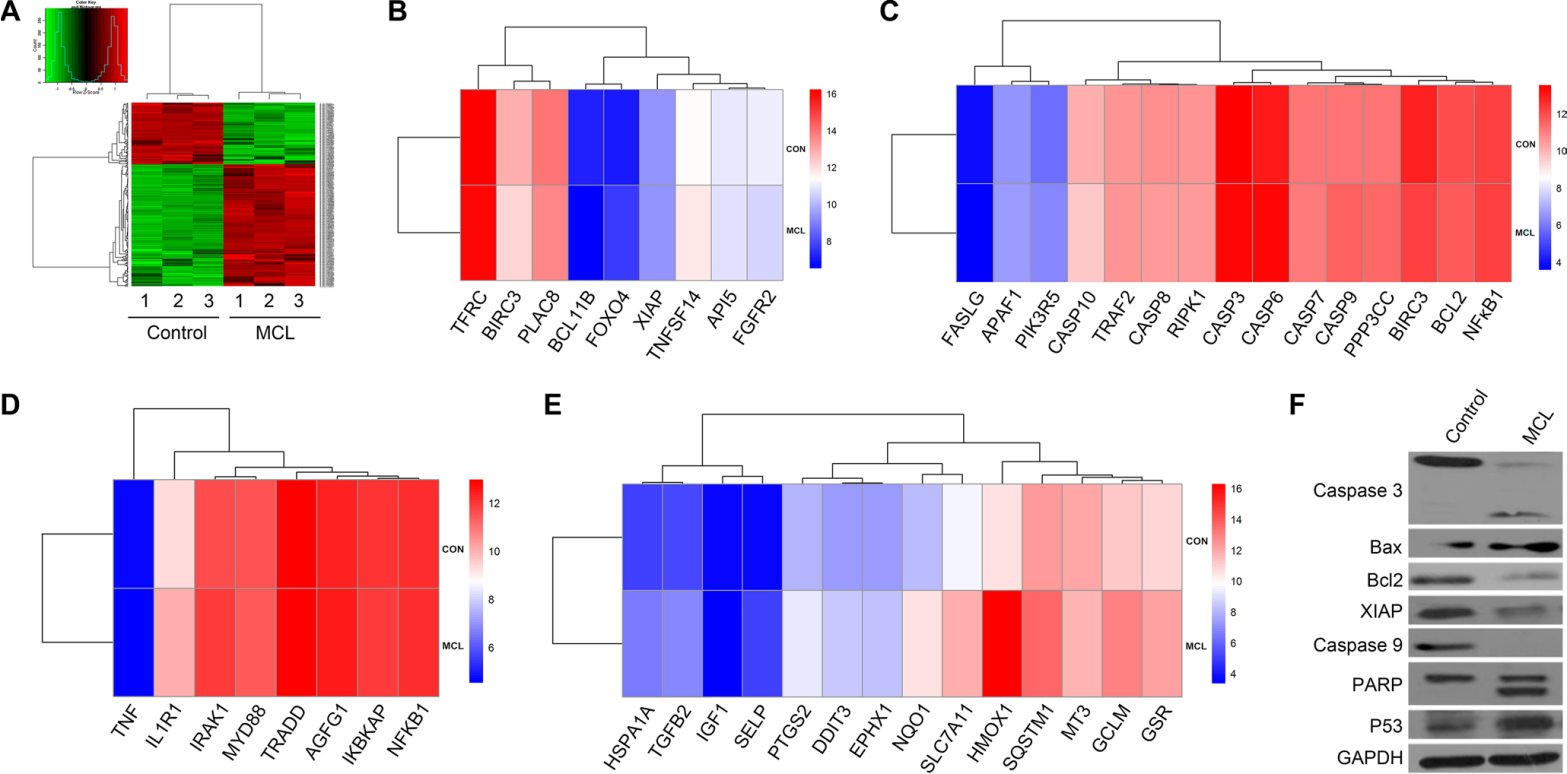
Transcriptome analysis of MCL-treated cells (**A**) Heat map analysis of microarray data showing hierarchical clustering of differentially expressed genes between untreated and MCL-treated KG1a cells. Three independent untreated and MCL-treated cell lines were analyzed. (**B**) Heat map demonstrating the differences in the anti-apoptotic transcriptional signature between control and MCL-treated cells. (**C**) Heat map demonstrating the differences in the pro-apoptotic transcriptional signature between control and MCL-treated cells. (**D**) Heap map demonstrating the differences in the NF-κB transcriptional signature between control and MCL-treated cells. (**E**) Heap map demonstrating the differences in the ROS transcriptional signature between control and MCL-treated cells. (**F**) Western blot analysis of apoptosis-associated proteins in Control and MCL-treated KG1a cells. GAPDH was used as a loading control.

To analyze the molecular mechanisms by which MCL induced apoptosis, microarray gene expression profiling of MCL-treated KG1a cells was performed. KG1a cells exhibit an LSC-like phenotype with high levels of CD34 expression (98.6%) and low levels of CD38 expression (24.6%). After MCL treatment, the expression of apoptosis-related genes was found to be altered at least two-fold (Figure 3A). In particular, several anti-apoptosis-related genes were down regulated, including *API5*, *FGFR2*, *TNFSF14*, *TFRC*, *XIAP*, *BIRC3*, and *PLAC8* compared to control (Figure 3B). Conversely, pro-apoptosis-associated genes, including *RIPK1*, *Bcl2*, *APAF1*, *PI3KR5*, *CASP9*, and *PPP3CC*, were up regulated compared to untreated cells (Figure 3C). To further verify the transcript profiling results, a Western blot analysis of KG1a cells and primary AML specimens was performed. Levels of the apoptosis-related protein, Bax, and phosphorylated p53 both increased, while levels of the anti-apoptosis proteins, Bcl-2 and XIAP, were significantly decreased (Figure 3F). Treatment with MCL also induced increased cleavage of caspase-3, caspase-9, and PARP, all of which are proteins that have been found to be associated with active apoptosis (Figure 3F).

NF-κB signaling and intracellular levels of ROS can vary between HSPCs and LSPCs. Therefore, the genes associated with these signaling pathways were analyzed. NF-κB-related targets, including *IL1R1*, *AGFG1, IRAK1*, *NF-κB1*, and *NF-κB1A* were found to be differentially expressed upon MCL treatment (Figure 3D). Additionally, the expression of genes related to ROS production and oxidative responses, including *GSR GCLM, HMOX1, SQSTM1, SLC7A11, EPHX1, DDIT3, PTGS2,* were up regulated (Figure 3E). Taken together, these data suggest that MCL may contribute to apoptosis through the NF-κB and ROS pathways.

### MCL inhibits NF-κB activity

**Figure 4:**
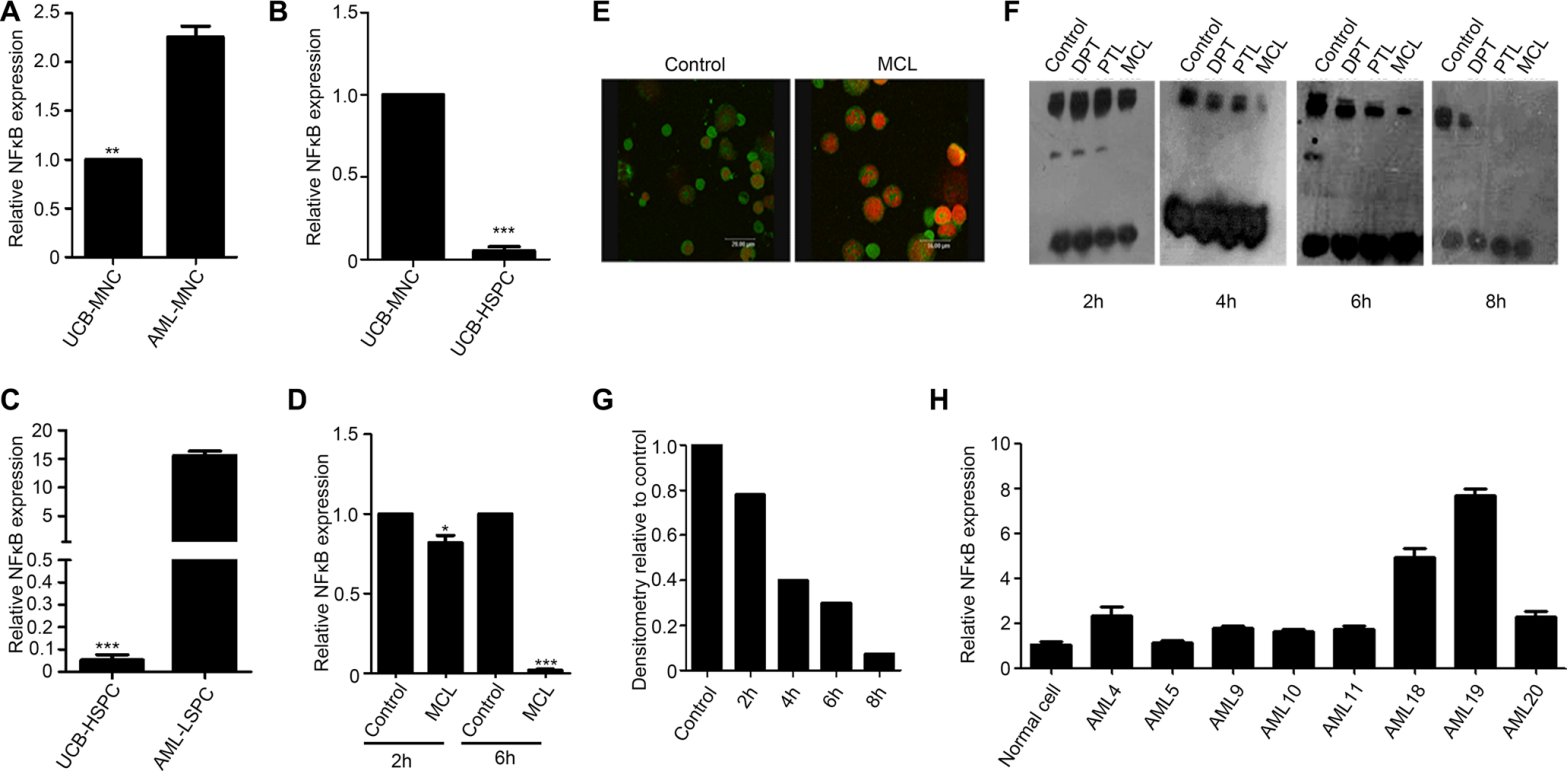
MCL inhibits NF-κB expression and activity in leukemic cells (**A**) Relative NF-κB expression in mononuclear cells isolated from human umbilical cord blood or primary AML specimens. Expression was normalized to GAPDH.(b) Relative NF-κB expression in mononuclear and HSPCs isolated from human umbilical cord blood. Expression was normalized to GAPDH. (**B**) Relative NF-κB expression in HSPCs isolated from umbilical cord blood and LSPCs isolated from primary AML specimens. Expression was normalized to GAPDH. (**C**) Relative NF-κB expression in AMI sample treated with 10 μM MCL after 2 h and 6 h. Control represents untreated cells. Expression was normalized to GAPDH. (**D**) Immunofluorescence assays detected cellular localization of NF-κB in bone marrow cells isolated from the AML mouse. The P65 subunit is shown in green and the nuclei are shown in red. (**E**) EMSA assay exhibiting NF-κB binding to DNA at various time points after primary AML cells were treated with 10 μM MCL. Control represents untreated cells. (**F**) Quantitative densitometry of gel shifts from (f) relative to control cells. (**G**) MNCs-mononuclear cells, UCB-umbilical cord blood, HSPCs-hematopoietic stem/progenitor cells, and LSPCs-leukemic stem/progenitor cells. (**H**) The gene expression of NF-κB in AML specimens compared to normal cells.

We next examined whether MCL exerts a direct effect on *NF-κB* expression. When primary AML MNCs were compared with normal MNCs obtained from umbilical cord blood, two-fold higher levels of *NF-κB* were detected (Figure 4A). In addition, a significant 15-fold increase in *NF-κB* expression was detected in LSPCs compared to MNCs isolated from the same AML specimens (Figure 4C). Conversely, *NF-κB* expression was significantly reduced in normal HSPCs compared to normal MNCs isolated from umbilical cord blood (Figure 4B; 10-fold). A change in *NF-κB* levels was not observed within 2 h of MCL treatment in KG1a cells, yet these levels were markedly reduced after 6 h of treatment (Figure 4D). Taken together, these findings suggested that *NF-κB* is selectively induced in LSCs, yet is significantly suppressed in response to MCL treatment. Thus, down regulation of NF-κB signaling may be required for MCL-induced apoptosis.

To further elucidate how MCL regulates NF-κB activity and function, cellular localization of NF-κB was examined in mice treated with DMAMCL. Within 3 h treatment, the NF-κB P65 subunit was observed to localize to the cytoplasm (Figure 4E). To investigate the effects of MCL treatment on the DNA-binding ability of NF-κB, electrophoretic mobility shift assay (EMSA) assays were performed. NF-κB binding to DNA was not significantly affected within 2 h of MCL treatment, yet binding of NF-κB to DNA was not detected after 8 h of MCL treatment (Figure 4F). When quantitative densitometry of the gel shifts was performed, MCL treatment was found to reduce the DNA binding ability of NF-κB in a time-dependent manner (Figure 4G). We further analyzed the expression of NF-κB in these specimens (Figure 4H), MCL exhibited better cytotoxicity in specimens with higher NF-κB expression. Collectively, these results provide supporting evidence that MCL-induced apoptosis may be mediated by inhibition of NF-κB expression and activity.

### Generation of intracellular ROS mediates MCL-induced apoptosis

**Figure 5:**
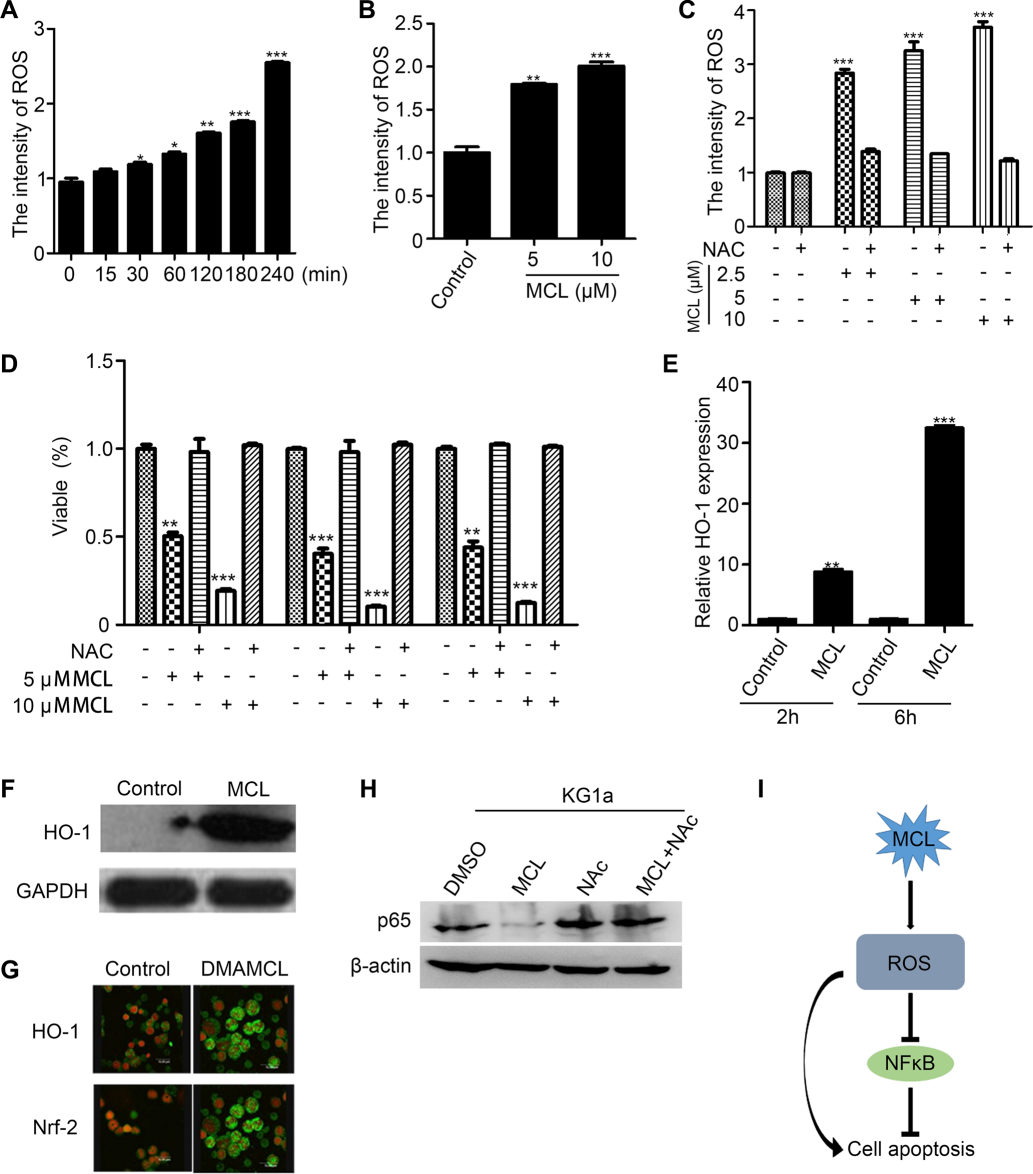
Generation of intracellular ROS promotes MCL-induced apoptosis (**A**) ROS levels in primary AML cells treated with 10 μM MCL at various time points over 240 min as measured by DCF-DA fluorescence. (**B**) ROS levels in primary CD34^+^ LSPCs treated with MCL for 1 h. (**C**) ROS levels in primary AML cells co-treated with MCL and NAC. (**D**) Percentage of viable LSPCs pretreated with NAC and then exposed to MCL. (**E**) Relative HO-1 expression in primary AML cells treated with 10 μM MCL after 2 h and 6 h. Control represents untreated cells. Expression was normalized to GAPDH. (**F**) Western blot analysis of HO-1 protein expression in primary AML cells treated with 10 μM MCL for 6 h. GAPDH was used as a loading control. (**G**) Immunofluorescence assays that show cellular localization of HO-1 and Nrf2 in bone marrow cells isolated from AML mice. HO-1/Nrf2 proteins are shown in green and nuclei are shown in red. (**H**) Western blot analysis of p65 protein expression with or without ROS inhibitor NAC. (**I**) Mechanism of MCL in inducing cell apoptosis was summarized.

The reactive oxidative state of MCL-treated AML cells was examined to determine if MCL-induced apoptosis is facilitated by increased generation of intracellular ROS. A flow cytometry analysis of primary AML MNCs and LSPCs indicated that peroxides were generated within 30 min of MCL treatment, and the levels continued to increase in a time- and dose-dependent manner (Figure 5A and 5B). In contrast, co-treatment of cells with MCL and the glutathione precursor, N-acetyl cysteine (NAC), prevented ROS production in primary AML MNCs (Figure 5C). Furthermore, pretreatment of LSPCs with NAC abrogated MCL-induced apoptosis (Figure 5D). A subsequent analysis of oxidative stress response mechanisms showed that *HO-1* gene expression increased approximately 8-fold after 2 h of MCL treatment in AML sample compared to untreated cells, and a 30-fold increase was detected after 6 h of treatment (Figure 5E). Accordingly, HO-1 protein levels significantly increased within 6 h of MCL treatment (Figure 5F), and immunofluorescence experiments further confirmed that expression of HO-1 and Nrf-2 both increased within 6 h of MCL treatment (Figure 5G). In combination, these results indicate that MCL exerts an apoptotic effect by increasing cellular oxidative stress. Many studies have shown that ROS mediated NF-κB cell signal pathway. To further analyze the relationship between ROS and NF-κB, a rescue experiment was conducted. As Figure 5H showed, the expression of p65 was reduced in response to MCL treatment. Nevertheless, co-treatment of cells with MCL and N-acetyl cysteine (NAC), an inhibitor of ROS, rescued the expression of p65. This result suggested that ROS could inhibit the expression of NF-κB to some extent. Furthermore, we summarized the mechanism of MCL. As shown in Figure 5I, MCL could induce cell apoptosis directly through the accumulation of ROS, while the accumulation of ROS could inhibit the activity of NF-κB.

## DISCUSSION

The presence of small populations of LSPCs that could replenish the malignant cell pool is proposed to be a leading reason for treatment failure and leukemia relapse [26, 27]. LSPCs also play an important role in driving chemo-resistance in patients [26]. Significant contributions by LSPCs are increasingly being realized, and efforts have been made to therapeutically target this stem cell niche as a strategy for the treatment of leukemia [7].

In our previous studies, we found that MCL was selectively cytotoxic to primary AML LSPCs, and detected the conversion of the pro-drug DMAMCL to MCL in plasma [24]. Therefore, in the present study, the effects of MCL on LSPCs *in vivo* were examined. MCL was found to exert selective and potent cytotoxic effects on LSPCs from primary AML samples, yet did not significantly affect normal MNCs and HSPCs. Furthermore, MCL was found to inhibit the capacity of LSPCs to form colonies, suggesting that MCL mediates a cytotoxic effect on LSPCs. After the treatment of AML sample with MCL for 18 h *in vitro*, a significant reduction in the engraftment of NOD/SCID mice was observed. The results from our NOD/SCID mouse model assays also demonstrated that various concentrations of MCL led to a significant increase in survival compared with ADR treatments. Collectively, our findings indicate that MCL could exert a strong cytotoxic effect on LSPCs, and MCL may represent a promising drug candidate for the treatment of leukemia.

Based on our transcriptome profiling of MCL-treated KG1a cells, the mechanisms by which MCL selectively targets LSPCs appear to be associated with activation of ROS and NF-κB signaling. LSPCs require a hypoxic environment for survival, and an increase in intracellular ROS can lead to DNA damage and the activation of apoptotic pathways. In the present study, we found that intracellular ROS in primary AML cells markedly increased within just 30 min of MCL treatment, and this effect was both time- and dose-dependent. We also found that the level of ROS was increased when LSPCs were treated with MCL, while ROS-induced apoptosis of LSPCs could be prevented with NAC pretreatment. The expression of HO-1, which is closely related to levels of intracellular ROS, was also up-regulated in response to MCL treatment and treatment with DMAMCL, with the latter accompanied by increased Nrf-2 as well. Both HO-1 and Nrf-2 are important transcription factors that regulate the production of ROS.

In addition to inducing intracellular ROS production, MCL was also found to inhibit NF-κB activity. NF-κB plays a key role in the survival of LSPCs. Expression of *NF-κB* in HSPCs was low, while could be significantly induced in LSPCs. Therefore, the expression and activity status of NF-κB may represent distinguishing features of LSPCs compared with HSPCs, and NF-κB may serve as a potential therapeutic target for the selective elimination of LSPCs. As previously reported, PTL [19], DMAPT [20], fenretinide [28], and niclosamide [29] also induce the apoptosis of LSPCs by inhibiting the NF-κB pathway. However, inhibition of NF-κB itself was not sufficient to induce robust apoptosis [30]. Nonetheless, in-activation of NF-κB may sensitize primary AML cells to increased intracellular levels of ROS and enhanced apoptosis [19].

A mutual interaction between ROS and NF-κB exists. ROS may negatively regulate NF-κB signaling above a certain threshold and was found to have an inhibitory effect on NF-κB activity [31–33]. Previous study has shown that ROS also mediated the role of NF-κB signal pathway in cells [34]. Our results proved that MCL may inhibit the NF-κB signaling activity by promote the accumulation of intracellular ROS. Meanwhile, this result provided some evidences that the accumulation of ROS activated NF-κB pathway.

In conclusion, the present findings demonstrate that MCL has the capacity to eliminate LSPCs, while with no substantial effects on normal HSPCs. Consequently, MCL was considerably effective at alleviating leukemic cell engraftment in the human AML mouse model. Mechanistically, the effects of MCL appear to be mediated by inhibition of NF-κB and increased ROS production. Therefore, further investigations into application of MCL for clinical treatment of AML are needed. Detailed mechanisms underlying MCL function and compound optimization should also be explored in future studies.

## MATERIALS AND METHODS

### Cell isolation, culture, and MCL compound synthesis

Primary human AML samples were obtained from volunteer donors at the Institute of Hematology & Blood Diseases Hospital. Human cord blood samples were obtained from volunteer donors at the Maternity Hospital. Mononuclear cells were isolated from each sample using Ficoll-Plaque density gradient separation and were cryopreserved in 90% fetal bovine serum (FBS)/10% dimethylsulfoxide (DMSO). Cells were cultured in serum-free Iscove's Modified Dulbecco's Medium (IMDM) for 1 h before the addition of MCL compounds that were synthesized by the Chenyue lab at Nankai University, China. All treatments were performed in triplicate. Human leukemia cell lines KG1a, HL60, K562, HL60/ADR and K562/A02 were obtained from State Key Laboratory of Experimental Hematology (Tian Jin, China). Then these cells were cultured in 1640 medium, supplemented with 10% fetal bovine serum at 37°C, 5% CO_2_.

### Mouse model of human AML

Initially, NOD/SCID immune-deficient mice were irradiated with 280 centigray (cGy). Then, 6–10 h later, the mice received a tail vein injection of primary AML mononuclear cells in a final volume of 0.2 ml PBS. Eight weeks after this cell transplantation, DMAMCL (50 mg/Kg) was administrated into the mice orally every other day. After 7 times the administration were stopped. The chemotherapy drug, adriamycin (ADR), were also administered every two days via tail vein at a dose of 1 mg/Kg for 4 times. Meanwhile, the survival was assessed and graphed by Kaplan-Meier plot. For immunofluorescence experiments, the mice received a single oral dose of 100 mg/Kg DMAMCL for 3 h and then were sacrificed.

### MTT cell proliferation assay

Briefly, leukemia cells were seeded in 24 plates (1 × 10^4^ cells/well). Cells were treated with varying concentrations of MCL for 72 h and optical density (OD) values were determined at 570 nm. IC_50_ values were calculated by using GraphPad Prism 5 (Graph Pad, San Diego, CA).

### Apoptosis assay

After 18 h of treatment with MCL, normal and AML samples were stained with CD34-allophycocyanin (APC) and CD38-PE.cy7 antibodies for 30 min. Cells were then washed with cold phosphate-buffered saline (PBS), resuspended in 100 μLof binding buffer, and incubated with Annexin-V-fluorescein isothiocyanate (FITC) and propidium iodide (PI) for 15 min. Samples were analyzed with a BD LSRII flow cytometer (BD Biosciences, New Jersey, America).

### Methylcellulose colony-forming cell assay

Mononuclear cells from primary AML specimens or umbilical cord blood were cultured in serum-free IMDM for 18 h in the presence or absence of 5 μM and 10 μM MCL. Treated cells were plated at a concentration of 2 × 10^5^ cells/ml in Methocult H4434 in 24 plates. Colonies were scored after 7–12 d. All treatments were performed in triplicate.

### Immunofluorescence and confocal microscopy

Cytocentrifuge preparations of bone marrow cells from AML mice were mounted onto glass slides and were left to dry overnight. Cells were then fixed with FAB Fluid for 1 min and were permeabilized with 0.5% Triton X-100 for 12 min. Cells were incubated in blocking buffer [10% FBS/0.1% Tween 20 in 1× PBS (pH 7.4)] and stained with rabbit polyclonal anti-p65 (C-20), anti-Nrf-2 (C-20) (Santa Cruz Biotechnology, Santa Cruz, CA), or anti-HO-1 (H-105; GeneTex Inc., San Antonio, TX) antibodies in 0.5% Triton X-100 for 2 h at RT. Cells were then washed and stained with goat anti-rabbit Alexa Fluor 488 (Invitrogen) secondary antibody for 30 min. Finally, nuclei were stained with TO-PRO-3 for 10 min and images were obtained using a 40 × objective.

### Electrophoretic mobility shift assay (EMSA)

EMSA was performed as described. Briefly, 4 μg of each nuclear extract was incubated with 1 μg of poly-d(I-C) and 400 fmol biotinylated NF-κB probe in 1X binding buffer and 2.5% glycerol for 25 min at RT. Protein/DNA complexes were resolved on a native polyacrylamide gel in 0.5% Tris/borate/EDTA (TBE) buffer at 4°C. After 2 h, the complexes were transferred to a nylon membrane for 1 h. Transferred DNA was cross-linked to the membrane and biotin-labeled DNA was detected by chemiluminescence.

### Quantitative RT-PCR

Using SYBR Green reagents (Takara), mRNA expression was assessed. The primer sequences used included: HO-1 forward: 5′-TGT GGT ACA GGG AGG CCA TCA CC -3′; HO-1 reverse: 5′-CAG GAT TTG TCA GAG GCC CTG AAG G-3′; Nrf-2 forward: 5′-TCA CCA TCT CAG GGG CAG -3′; Nrf-2 reverse: 5′-CAA CAT ACT GAC ACT CCA ATG C-3′; NF-ĸB forward: 5′-GGA GGC ATG TTC GGT AGT GG-3′; NF-ĸB reverse: 5′-CCC TGC GTT GGA TTT CGT G-3′. And GAPDH forward: 5′-AGGTCGGTGTGAACGGATTTG-3′; GAPDH reverse: 5′-GGGGTCGTTGATGGCAACA-3′. And the expression data was analyzed with Delta Ct.

### ROS production

Cells treated with MCL were collected, washed with PBS, and incubated with 10 nM 2′, 7′-Dichlorofluorescin diacetate (DCF-DA) (Beyotime, China) for 30 min at 37°C. Samples were then washed twice with PBS and the intensity of FITC fluorescence was measured by flow cytometry (BD LSRII flow cytometer, BD Biosciences).

### Microarray transcriptional profiling

KG1a cells were treated with 10 μM MCL for 8 h. Each sample was performed in triplicate. Cells were then frozen at −20°C and a gene chip assay was performed by Shanghai Biotechnology Company (Shanghai, China). KG1a cells were collected for microarray analysis using Affymetrix Human Genome U133 plus 2.0 Array (Affymetrix). The expression data are available at National Center for Biotechnology Information Gene Expression Omnibus with accession number GSE76384.

### Western blotting assay

KG1a cells and AML sample cells were treated with 10 μM MCL for 8 h before protein lysis buffer was added to each set of cells for 30 min on ice. Then, centrifuged, and total protein concentrations were determined using BCA reagent. Each protein extract sample (50 μg) was separated on a 12% gel, transferred to a nitrocellulose membrane, and incubated with primary antibodies raised against Bax, Bcl-2, PARP, caspase-3, caspase-9, P53, P-P53, XIAP, and GAPDH (Cell signaling technology, Boston, America) at 4°C overnight. The membranes were washed with PBS three times before being incubated with the appropriate anti-HRP secondary antibodies (Cell signaling technology, Boston, America) (1:2000) at RT. After 2 h, bound antibodies were detected with an ECL-Plus Kit (Thermo Scientific, Rockford, America).
